# Supplementary material for: Solving Color Reproducibility between Digital Devices: A Robust Approach of Smartphones Color Management for Chemical (Bio)Sensors
Source: Biosensors (Basel). 2022 May 17;12(5):341. doi: 10.3390/bios12050341 (PMC9139083; doi:10.3390/bios12050341)
Supplement: Supplementary file 1 [file biosensors-12-00341-s001.zip › biosensors-1674657-supplementary.pdf]

biosensors

Supplementary Materials

# Solving color reproducibility between digital devices. A robust approach of Smartphones color management for chemical (bio)sensors

**Pablo Cebrián Aznárez [1], Leticia Pérez-Sienes [2], Isabel Sanz-Vicente [1], Ángel López-Molinero [1], Susana de Marcos [1], Javier Galbán* [1]**

[1] Nanosensors and Bioanalytical Systems group (N&SB), University of Zaragoza, Instituto de Nanociencia de Aragón (INA), and Instituto de Carboquímica (ICB-CSIC), Zaragoza, Spain.; e-mail@e-mail.com
[2] Complex Systems Group, Polytechnic University of Madrid, ETSI Agronomy, Food and Biosystems, 28040 Madrid, Spain; e-mail@e-mail.com
* Correspondence: jgalban@unizar.es;

**Citation:** *Biosensors* **2022**, *12*, 341. https://doi.org/10.3390/bios12050341

Received: 25 March 2022
Accepted: 9 May 2022
Published: 17 May 2022

**Publisher's Note:** MDPI stays neutral with regard to jurisdictional claims in published maps and institutional affiliations.

**Copyright:** © 2022 by the authors. Licensee MDPI, Basel, Switzerland. This article is an open access article distributed under the terms and conditions of the Creative Commons Attribution (CC BY) license (https://creativecommons.org/licenses/by/4.0/).

## Supplementary Material 1:

**Bayer mosaic filter [1]:** It consists of a color filter array which allows the photodiodes to receive only part of the light (red, green and blue wavelengths or RGB channels) and thus be able to interpret the color. The filter pattern is half green, one quarter red and one quarter blue, being the smallest homogeneous unit in this array called pixel, which enables to create a digital image (Figure S1[2]). This light-caption interpretation is based on the same principle as the human eye sees color, by means of additive reproduction, which means that every color in the visible spectrum can be generated as the mixture of red, green and blue colors or what is known as the RGB color system. In this way, depending on the amount of red, green and blue light that the pixels capture, it will be the result of the final color of the digital image.

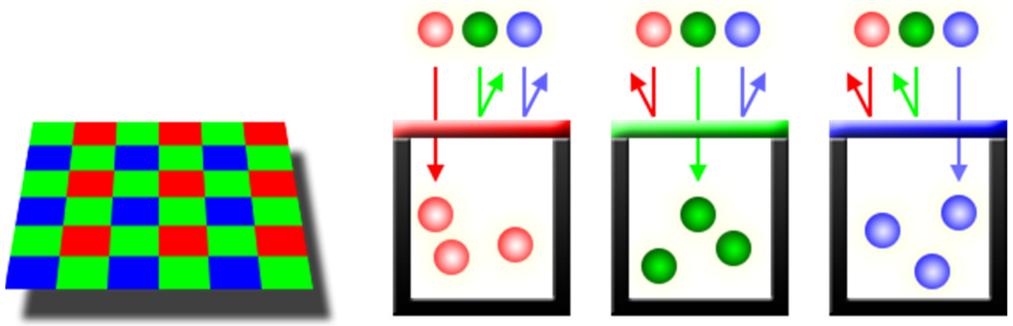

**Figure S1.** The Bayer arrangement of color filters on the pixel array of an image sensor. The right part of the image shows the side-view of color photosites.

## Supplementary Material 2:

**sRGB color space transformation [3,4]:** First, the information recollected from the companded RGB channels of the sensor (denoted with upper case ($R_0$, $G_0$, $B_0$), or generically V) are divided by 255. These values depend of the light that the sensor captures and stablishes how colors will be shown in a screen, so they have to be transformed from an analogical signal ($R_0$, $G_0$, $B_0$) to a digital signal (denoted with lower case ($r_0$, $g_0$, $b_0$), or generically v). This can be expressed as follows:

$$\boldsymbol{V} \in \{\boldsymbol{R_0}, \boldsymbol{G_0}, \boldsymbol{B_0}\}$$

$$v \in \{r_0, g_0, b_0\}$$

The same operation is performed on all three channels, but the operation depends on the companding function associated with the RGB color system used, in our case a JPEG photography has associated the sRGB system (Equation S1).

$$v = \begin{cases} V/12.92 & if\ V \leq 0.04045 \\ ((V + 0.055)/1.055)^{2.4} & otherwise \end{cases} \quad \text{(S1)}$$

**Supplementary Material 3:**

**Color models:** A color model or color space is defined as [5] "*the digital representation of possibly contained colors*" or as [6] "*the way that we can recognize color, where human can visualize color through its attributes such as; hue, and brightness*". Depending of the area in which it is going to be applied we can distinguish:

**Table S1.** Application Areas of Color Models [5]

| Color Model | Application Area |
|---|---|
| Munsell | Human visual system |
| RGB | Computer graphics, Image processing, Analysis, Storage |
| CMYK | Printing |
| YIQ, YUV | TV broadcasting, Video system |
| YCbCr | Digital video |
| HIS, HSV, HSL | Human visual perception, Computer graphics, processing, Computer Vision, Image Analysis, Design image, Human vision, Image editing software, Video editor |
| CIE XYZ, CIE Luv, CIE Lab | Evaluation of color difference, Color matching system, advertising, graphic arts, digitized or animated paintings, multimedia products |

**Supplementary Material 4**

You can access this software in the following link
https://github.com/lpsienes/color_reproducibility_for_smartphones

**Supplementary Material 5:**

**RGB mathematical transformation to CIE Lab [3,4]:**

- First, the initial RGB values ($R_0$, $G_0$, $B_0$) have to be transformed, as explained in the *"sRGB color space transformation"*(Supplementary Material), to $r_0$, $g_0$, $b_0$. The 3D interpretation of the RGB color system is shown in Figure S2.a.
- Then, these new values are transformed into an intermediate color system called CIE XYZ (Figure S2.b), a wavelength dependent color representation system created by

CIE that maps out all the colors the human-eye can see (Equation S2). The matrix used for this transformation is stablished for the transformation of the sRGB system to CIE XYZ using a $D_{65}$ illuminant as reference:

$$\begin{bmatrix} X \\ Y \\ Z \end{bmatrix} = \begin{bmatrix} 0.41245 & 0.35757 & 0.18043 \\ 0.21267 & 0.71515 & 0.07217 \\ 0.01933 & 0.11919 & 0.95030 \end{bmatrix} \begin{bmatrix} r_0 \\ g_0 \\ b_0 \end{bmatrix} \quad \text{(S2)}$$

Another commonly used color system is the CIE xy (Figure S2.c), a projection of the XYZ system where only chromaticity is contemplated for a 2D graphical representation (Equations S3 and S4).

$$x = \frac{X}{X+Y+Z} \quad \text{(S3)}$$

$$y = \frac{Y}{X+Y+Z} \quad \text{(S4)}$$

- As CIE Lab color system concerns, it was developed specifically to serve as a reference. This system, unlike others mentioned, is perceptually linear due to the sphericity of the color system, that is, it assumes that a change of the same amount in color value must produce a change of the same visual importance. That is why is used as to quantify the differences between two colors (reference and measurement) and to establish tolerance limits. This system is made up of three coordinates: L (Luminosity - Z axis), a (amount of red or green – X axis) and b (amount of yellow or blue – Y axis). Its graphical representation is a sphere of radius 100 whose Z axis represents different intensities of the reference illuminant used. To relate the CIE Lab system (Figure S2.d) to the XYZ, illuminant reference values are needed (Xr, Yr, Zr). For the D65 illuminant those coordenates are: Xr = 0.95047, Yr = 1.00000, and Zr = 1.08883 [28,29]. The conversion is as follow (Equations S5-S11):

$$L = 116 f_y - 16 \quad \text{(S5)}$$

$$a = 500\left(f_x - f_y\right) \quad \text{(S6)}$$

$$b = 200\left(f_y - f_z\right) \quad \text{(S7)}$$

Where:

$$f_x = \begin{cases} \sqrt[3]{x_r} & if\ y_r > \epsilon \\ \frac{\kappa x_r + 16}{116} & otherwise \end{cases} \quad \text{(S8)}$$

$$f_y = \begin{cases} \sqrt[3]{y_r} & if\ y_r > \epsilon \\ \frac{\kappa y_r + 16}{116} & otherwise \end{cases} \quad \text{(S9)}$$

$$f_z = \begin{cases} \sqrt[3]{z_r} & if\ y_r > \epsilon \\ \frac{\kappa z_r + 16}{116} & otherwise \end{cases} \quad \text{(S10)}$$

$$x_r = \frac{X}{X_r}; y_r = \frac{Y}{Y_r}, z_r = \frac{Z}{Z_r} \quad \text{(S11)}$$

$$\epsilon = \begin{cases} 0.008856 & Actual\ CIE\ standard \\ \dfrac{216}{24389} & Intent\ of\ the\ CIE\ standard \end{cases}$$

$$\kappa = \begin{cases} 903.3 & Actual\ CIE\ standard \\ 24389/27 & Intent\ of\ the\ CIE\ standard \end{cases}$$

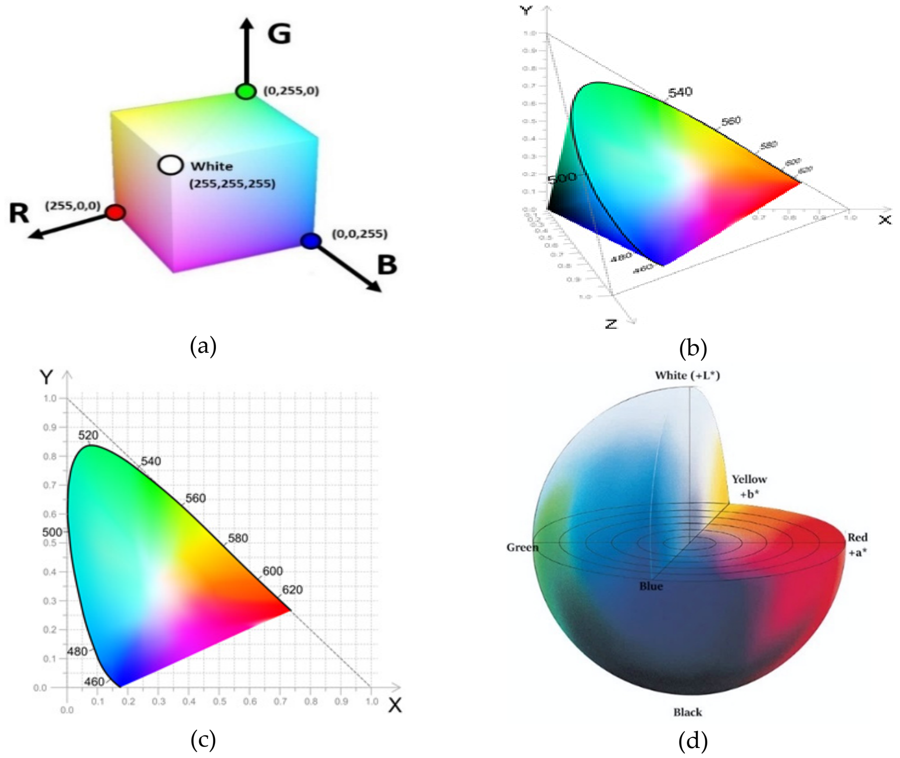


**Figure S2.** Different color spaces and their numerical representation. (a) RGB color space. (b)CIE XYZ color space. (c) CIE xy color space.(d) CIE Lab color space.

**Supplementary Material 6:**

**CIEΔE2000 [3,4,7] and Parametric Factors using CIEΔE2000 [8]:** The Delta E value is a standard measurement, created by the Commission Internationale de l′Eclairage (International Commission on Illumination), that quantifies the difference between two colors, or color accuracy, that appears on a screen. The equations (S12-S35) to quantify CIEΔE2000 are:

$$\Delta E = \sqrt{(A)^2 + (B)^2 + (C)^2 + R_T(B)(C)} \quad \text{(S12)}$$

$$* A = \frac{\Delta L^{'}}{K_L S_L}; B = \frac{\Delta C^{'}}{K_C S_C}; C = \frac{\Delta H^{'}}{K_H S_H}\ ;\ K_L = 1;\ K_C = 1;\ K_H = 1 \quad \text{(S13)}$$

Where:

$$L^{'} = (L_1 + L_2)/2 \quad \text{(S14)}$$

$$C_1 = \sqrt{{a_1}^2 + {b_1}^2};\ C_2 = \sqrt{{a_2}^2 + {b_2}^2} \quad \text{(S15)}$$

$$C = (C_1 + C_2)/2 \quad \text{(S16)}$$

$$G = \frac{1}{2}\left(1 - \sqrt{\frac{C^7}{C^7 + 25^7}}\right) \tag{S17}$$

$$a_1^{'} = a_1(1+G); a_2^{'} = a_2(1+G) \tag{S18}$$

$$C_1^{'} = \sqrt{(a_1^{'})^2 + (b_1)^2} \tag{S19}$$

$$C_2^{'} = \sqrt{(a_2^{'})^2 + (b_2)^2} \tag{S20}$$

$$\bar{C}^{'} = (C_1^{'}/C_2^{'})/2 \tag{S21}$$

$$h_1^{'} = \begin{cases} \arctan(b_1/a_1^{'}) & if\ arctan(b_1/a_1^{'}) \geq 0 \\ \arctan(b_1/a_1^{'}) + 360^{\circ} & otherwise \end{cases} \tag{S22}$$

$$h_2^{'} = \begin{cases} \arctan(b_1/a_2^{'}) & if\ arctan(b_1/a_2^{'}) \geq 0 \\ \arctan(b_1/a_2^{'}) + 360^{\circ} & otherwise \end{cases} \tag{S23}$$

$$\bar{H}^{'} = \begin{cases} (h_1^{'} + h_2^{'} + 360^{\circ})/2 & if\left|h_1^{'} - h_2^{'}\right| > 180^{\circ} \\ (h_1^{'} + h_2^{'})/2 & otherwise \end{cases} \tag{S24}$$

$$T = 1 - 0.17\cos(\bar{H}^{'} - 30^{\circ}) + 0.24\cos(2\bar{H}^{'}) + 0.32\cos(3\bar{H}^{'}{'} + 6^{\circ}) - 0.20\cos(4\bar{H}^{'}{'} - 63^{\circ}) \tag{S25}$$

$$\Delta h^{'} = \begin{cases} h_2^{'} - h_1^{'} & if\left|h_1^{'} - h_2^{'}\right| \leq 180^{\circ} \\ h_2^{'} - h_1^{'} + 360^{\circ} & else\ if\left|h_1^{'} - h_2^{'}\right| > 180^{\circ}\ and\ h_2^{'} \leq h_1^{'} \\ h_2^{'} - h_1^{'} - 360^{\circ} & otherwise \end{cases} \tag{S26}$$

$$\Delta L^{'} = L_2 - L_1 \tag{S27}$$

$$\Delta C^{'} = C_2^{'} - C_1^{'} \tag{S28}$$

$$\Delta H^{'} = 2\sqrt{C_1^{'}C_2^{'}}\sin(\Delta h^{'}/2) \tag{S29}$$

$$S_L = 1 + \frac{0.015(L^{'} - 50)^2}{\sqrt{20 + (L^{'} - 50)^2}} \tag{S30}$$

$$S_C = 1 + 0.045\bar{C}^{'} \tag{S31}$$

$$S_H = 1 + 0.015\bar{C}^{'}T \tag{S32}$$

$$\Delta\theta = 30exp\left\{-\left(\frac{\bar{H}^{'} - 275^{\circ}}{25}\right)^2\right\} \tag{S33}$$

$$R_C = 2\sqrt{\frac{(\bar{C}^{'})^7}{(\bar{C}^{'})^7 + 25^7}} \tag{S34}$$

$$R_T = -R_c\sin(2\Delta\theta) \tag{S35}$$

**Table S2.** Parametric Factors using **CIEΔE$_{2000}$**

| $K_L$ = 1 (2 in our experiments), $K_C$ = $K_H$ = 1 under "reference conditions |
| --- |
| Illumination: $D_{65}$ source |
| Illuminance: 1000 lx |
| Observer: Normal color vision |
| Background field: Uniform, neutral gray with L = 50 |
| Viewing mode: Object |
| Sample size: Greater than 4 degrees |
| Sample separation: Direct edge contact |
| Sample color-difference magnitude: Lower than 5.0 ΔE |
| Sample structure: Homogeneous (Without texture) |

**Supplementary Material 7:**

**Comparation of 96 RAL Classic ® color samples:** Table S3 shows the ΔE values of 96 RAL color samples characterized with a CM-2600d Spectrophotometer. The samples that did not meet the JND criterion were discarded**.**

**Table S3.** Validation of 96 color samples with a CM-2600d Spectrophotometer.

| RAL | ΔE | RAL | ΔE | RAL | ΔE | RAL | ΔE |
|---|---|---|---|---|---|---|---|
| 1000 | 1.17 | 2012 | 2.04 | 4004 | 1.57 | 6000 | 1.09 |
| 1001 | 0.67 | 3000 | 1.16 | 4005 | 0.39 | 6001 | 1.41 |
| 1002 | 0.55 | ~~3001~~ [1] | ~~4.12~~ | 4006 | 0.74 | 6002 | 1.79 |
| ~~1003~~ [1] | ~~2.35~~ | 3002 | 2.06 | 4007 | 2.08 | 6003 | 1.34 |
| 1004 | 0.83 | ~~3003~~ [1] | ~~2.55~~ | 4008 | 1.42 | 6004 | 1.02 |
| 1005 | 1.60 | 3004 | 0.90 | 4009 | 0.90 | ~~6005~~ [1] | ~~2.49~~ |
| 1015 | 0.42 | ~~3005~~ [1] | ~~2.31~~ | 4010 | 0.69 | 6018 | 1.98 |
| 1016 | 0.56 | 3007 | 2.11 | 5000 | 1.74 | 6019 | 0.49 |
| 1017 | 1.15 | 3009 | 0.90 | 5001 | 1.66 | 6020 | 0.34 |
| 1018 | 1.25 | 3011 | 1.07 | 5002 | 0.25 | 6021 | 0.52 |
| 1019 | 1.29 | 3012 | 0.68 | ~~5003~~ [1] | ~~3.26~~ | 6022 | 0.55 |
| 1020 | 1.44 | 3013 | 0.98 | ~~5004~~ [1] | ~~4.13~~ | 6024 | 1.36 |
| 1033 | 2.09 | 3014 | 0.97 | 5012 | 0.81 | 6025 | 0.71 |
| 1034 | 1.30 | 3015 | 0.99 | 5013 | 1.09 | ~~6026~~ [1] | ~~3.12~~ |
| 1037 | 2.18 | ~~3016~~ [1] | ~~3.33~~ | 5014 | 1.05 | 6027 | 0.98 |
| 2000 | 2.26 | 3017 | 1.01 | 5015 | 1.19 | 6028 | 0.56 |
| 2001 | 1.28 | 3018 | 0.69 | 5017 | 1.64 | 6029 | 2.22 |
| ~~2002~~ [1] | ~~2.79~~ | ~~3020~~ [1] | ~~2.35~~ | 5018 | 0.63 | 6032 | 1.37 |
| 2003 | 1.12 | 3022 | 1.31 | 5019 | 2.23 | 7004 | 0.84 |
| 2004 | 1.64 | 3027 | 1.27 | ~~5020~~ [1] | ~~2.49~~ | 7005 | 0.70 |
| 2008 | 2.23 | 3031 | 1.14 | 5021 | 0.99 | 9010 | 0.83 |
| ~~2009~~ [1] | ~~2.52~~ | 4001 | 0.81 | 5022 | 1.19 | 9011 | 1.47 |
| ~~2010~~ [1] | ~~3.82~~ | 4002 | 1.02 | 5023 | 0.74 | 9016 | 1.54 |
| 2011 | 0.87 | 4003 | 0.89 | 5024 | 0.65 | ~~9017~~ [1] | ~~2.86~~ |

[1] CM-2600d Spectrophotometer Screening test (The red crossed-out samples had some imperfections on their surface so they did not meet the JND criterion. Theses samples were discarder).

**Supplementary Material 8:**

**Study of the RAL´s surface integrity:** As it can be appreciated in the images taken with the USB Dino-Lite AM2111 microscope (Figures S3.a and S3.b) the surface´s integrity is not the same and this will affect the color measurements.

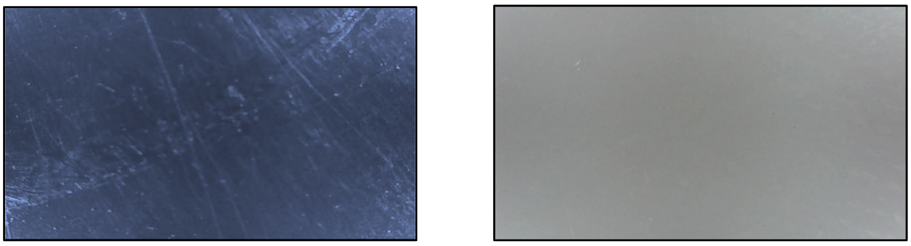

(a) (b)

**Figure S3.** Photographs of two RAL color sample´s surface taken with the Dino-Lite AM2111 Microscope. (a) RAL 5004 surface. Since the surface has deteriorated due to its use, the RGB values of this sample will be less accurate than in a sample with a

homogeneous surface. (b) RAL 7004 surface. The surface is more homogeneous compared with the RAL 5004 surface.

**Supplementary Material 9:**

**Comparation of 81 RAL Classic ® color samples:** Table S4 shows the ΔE values of 81 RAL color samples characterized with an HP Scanjet G2410 Flatbed Scanner. The samples that had an RGB coordinate equal to 0 or 255 did not meet the JND criterion and were discarded**.**

**Table S4.** Validation of 81 color samples with an HP Scanjet G2410 Flatbed Scanner

| RAL | ΔE | RAL | ΔE | RAL | ΔE | RAL | ΔE |
|---|---|---|---|---|---|---|---|
| 1000 | 0.89 | 2012 | 1.52 | 4004 | 1.49 | 6000 | 1.96 |
| 1001 | 0.33 | 3000 | 1.98 | 4005 | 2.13 | 6001 | 2.21 |
| 1002 | 1.72 | - | - | 4006 | 1.66 | 6002 | 1.02 |
| - | - | 3002 | 1.78 | 4007 | 2.24 | 6003 | 1.99 |
| ~~1004~~ [1] | ~~3.44~~ | - | - | 4008 | 1.76 | ~~6004~~ [1] | ~~1.40~~ |
| ~~1005~~ [1] | ~~3.79~~ | 3004 | 1.62 | 4009 | 2.29 | - | |
| 1015 | 1.15 | - | - | 4010 | 1.26 | 6018 | 1.14 |
| ~~1016~~ [1] | ~~7.48~~ | 3007 | 2.24 | 5000 | 2.25 | 6019 | 1.60 |
| ~~1017~~ [1] | ~~5.60~~ | 3009 | 0.41 | ~~5001~~ [1] | ~~2.04~~ | 6020 | 2.23 |
| ~~1018~~ [1] | ~~2.22~~ | 3011 | 1.91 | ~~5002~~ [1] | ~~1.09~~ | 6021 | 1.61 |
| 1019 | 2.21 | 3012 | 1.35 | - | - | 6022 | 1.68 |
| 1020 | 1.44 | 3013 | 2.19 | - | - | ~~6024~~ [1] | ~~2.68~~ |
| 1033 | 2.08 | 3014 | 2.17 | ~~5012~~ [1] | ~~3.22~~ | 6025 | 2.19 |
| 1034 | 2.28 | 3015 | 1.77 | 5013 | 0.93 | - | - |
| ~~1037~~ [1] | ~~2.99~~ | - | - | 5014 | 2.29 | 6027 | 2.22 |
| ~~2000~~ [1] | ~~4.39~~ | 3017 | 2.02 | ~~5015~~ [1] | ~~6.25~~ | 6028 | 1.77 |
| ~~2001~~ [1] | ~~1.81~~ | 3018 | 2.24 | ~~5017~~ [1] | ~~14.72~~ | ~~6029~~ [1] | ~~6.88~~ |
| - | - | - | - | ~~5018~~ [1] | ~~4.51~~ | 6032 | 1.31 |
| ~~2003~~ [1] | ~~2.19~~ | 3022 | 2.21 | ~~5019~~ [1] | ~~6.96~~ | 7004 | 1.40 |
| ~~2004~~ [1] | ~~6.45~~ | 3027 | 0.76 | - | - | 7005 | 2.24 |
| ~~2008~~ [1] | ~~2.34~~ | 3031 | 1.72 | ~~5021~~ [1] | ~~9.71~~ | ~~9010~~ [1] | ~~3.85~~ |
| - | - | 4001 | 2.24 | 5022 | 2.28 | 9011 | 2.26 |
| - | - | 4002 | 1.96 | 5023 | 1.71 | ~~9016~~ [1] | ~~1.65~~ |
| ~~2011~~ [1] | ~~1.13~~ | 4003 | 0.90 | 5024 | 0.68 | - | - |

[1] HP Scanjet G2410 Flatbed Scanner Screening test (The purpled crossed-out samples had an RGB coordinate equal to 0 or 255 so they did not meet the JND criterion. Theses samples were discarder).

**Supplementary Material 10:**

**Spectral Similarity Index method [9]:** A Spectral Similarity Index is a comparison method that, unlike others indexes used to predict the color rendering quality (CRI or CCT), is based upon the similarity of a light source´s spectral power emission at various wavelengths to a reference spectral power distribution (typically $D_{50}$), making it independent of camera sensitivities, since the spectral sensitivities of the cameras can vary widely from model to model. Figures S4a and S4b show a graph of the spectral power distribution of two light sources used (test) compared to a $D_{50}$ (reference) and the variance between them (blue-shaded area). The SSI concept relies on the difference between areas of both spectra. If this difference is small enough, the spectrum of the test source is effectively the same as the reference source and it will produce the same color measurements.

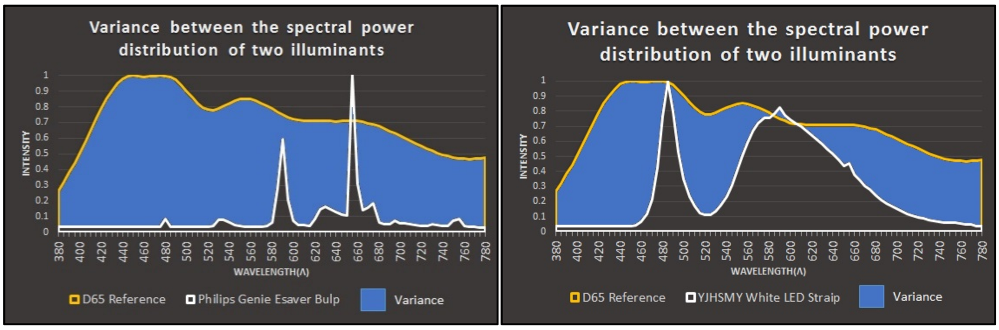


(a) (b)

**Figure S4.** Spectral Similarity Index of two different illuminants used in the *"lighting effect on colors of digital images taken with a Smartphone study"*. (a) Variance of a Philips Genie Esaver Bulp and a $D_{50}$ Illuminant. (b) Variance of a YJHSMY White LED Strip and a $D_{50}$ Illuminant.

The SSI value is scaled to a 100-point scale, were high scores mean there will probably be no issue reproducing colors, while lower scores indicate greater differences. The SSI value is always denoted with the reference illuminant used for comparison shown in [brackets]. Table S5 shows the comparison of the SSI values with the CIE$\Delta E_{2000}$, without correction (WOC) and with correction (WC), previously calculated.

**Table S5.** Comparison of the SSI of four illuminants with the CIE$\Delta E_{2000}$ values before (WOC) and after (WC) the correction method is applied.

| Illuminant | SSI [1] | $\Delta E_{WOC}$ | $\Delta E_{WC}$ | $\Delta E_{WOC} - \Delta E_{WC}$ |
|---|---|---|---|---|
| EGLO RGB LED Strip | 27 | 15.87 | 4.12 | 11.75 |
| Philips Genie Esaver Bulp | 31 | 13.30 | 3.76 | 9.54 |
| Philips Master TL-D Fluorescent | 52 | 8.33 | 2.98 | 5.35 |
| YJHSMY White LED Strip | 59 | 6.65 | 2.61 | 4.04 |

[1] The SSI reference illuminant is $D_{50}$

This comparison shows that a lower SSI of an illuminant increases the initial ΔE value of the measurements, which is consistent with the spectral differences between the reference illuminant and the one used. These SSI values allow to evaluate and choose which illuminant is better for color rendering, minimizing initial errors and improving the color corrections of the method proposed.

**Spectral Similarity Index Calculation [10]:** In designing SSI, the range of wavelengths to be included in its calculation was determined by the range of sensitivity of photographic film and multiple digital cinema and still cameras. The Spectral Power Distribution data are "binned" into 10 nm samples to accommodate small irregularities and measurement tolerances. The binned values are then weighted so that less emphasis is placed on the lower and upper wavelengths, where variances have less overall effect on color-rendering results. The values are smoothed using an additional weighting factor to reduce the effect of minor deviations. These mathematical adjustments were optimized using Fourier analysis and simplified using convolution for calculation purposes. A test luminaire's SSI value is computed as follows:

1) Specify test and reference source SPDs (at intervals not exceeding 5 nm).
2) Interpolate spectra to 1-nm increments from 375 nm to 675 nm (padding with zeroes if the test luminaire is not specified fully across that range).
3) Integrate spectra in 10-nm intervals from 380 to 670 nm.

4) Normalize to unity total power of test and reference sources by dividing each 10-nm sample by sum of all 10-nm samples for each source.
5) Calculate relative difference vector = ( normalized test source vector – normalized reference source vector) / (normalized reference source vector + 1/30 ).
6) Calculate weighted relative difference vector = relative difference vector * spectral weighting vector { 4/15, 22/45, 32/45, 8/9, 44/45, 1, 1, 1, 1, 1, 1, 1, 1, 1, 1, 1, 1, 1, 1, 1, 1, 1, 1, 1, 1, 1, 1, 1, 1, 11/15, 3/15 }.
7) Add zero to each end of weighted relative difference vector to have 32 values.
8) Convolve with {0.22, 0.56, 0.22} to create 30-element smoothed weighted relative difference vector.
9) Calculate vector magnitude = square root of sum of squares of elements of smoothed weighted relative difference vector.
10) SSI value = round (100 – 32 * vector magnitude).

**Supplementary Material 11:**

**Histogram, 3D surface plot and the effect of external illumination inside the light box:** In order to prove the problems associated to non-controlled lighting conditions, a random RAL color was selected from the chart (RAL 6018):

- First, a histogram study of the RAL 6018 RGB channels with/without the use of a light box was made. A histogram is a graphical representation of the frequencies of the RGB values on a selected area of an image. These graphs will show the distribution of the recollected data by the sensor and the symmetry of it. Figures S5a,S5b and S5c show the histograms of each RGB Channel value on the RAL 6018 color samples with and without a Light Box using the "YJHSMY White LED Strip Illuminant". The comparation of each of the RGB channels histograms, respectively, showed that the use of a light box reduces the uncertainty of the color measurements, obtaining more accurate results than the case of not using it due to the presence of other illuminants.

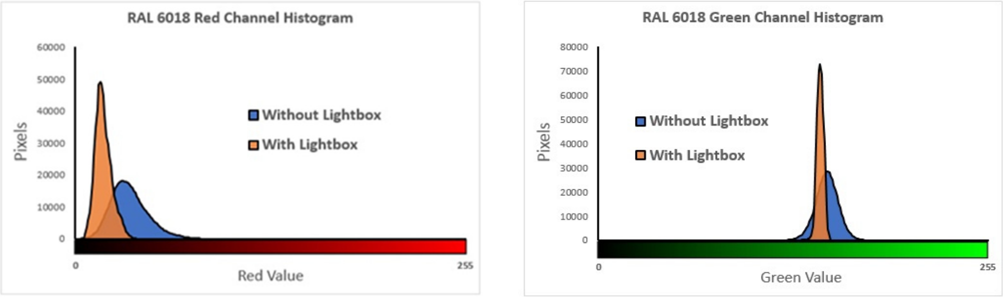


(a) (b)

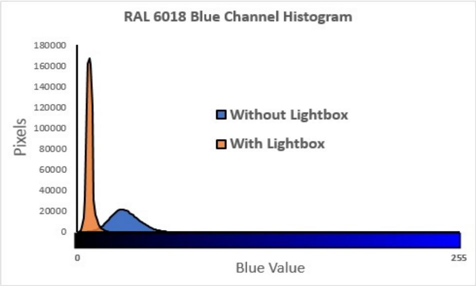


(c)

**Figure S5.** Histogram comparation of the RAL 6018 RGB channels with/without the use of a Light box. (a) Red channel histogram with/without the use of a Light box. (b) Green channel histogram with/without the use of a Light box. (c) Blue channel histogram with/without the use of a Light box

- Then, the 3D surface plot tool was used in order to evaluate the light distribution on the color sample´s surface. This tool shows the relative intensity of each pixel (RPI) (Equation S36) on a selected area of a digital image as a 3D color gradient graph. The RPI value is scaled to a 100-point scale, were a 100 value is colored in red, while a 0 value is colored in blue. As shown in Figure S6a and S6b there is not a homogeneous distribution of light in the selected area of the image, compared with Figures S6c and S6d, which show a more homogeneous distribution.

$$\mathbf{RPI} = \frac{\sqrt{\mathbf{R_0}^2+\mathbf{G_0}^2+\mathbf{B_0}^2}}{\sqrt{\mathbf{R_{0,max}}^2+\mathbf{G_{0,max}}^2+\mathbf{B_{0,max}}^2}} \cdot \mathbf{100} \quad \text{(S36)}$$

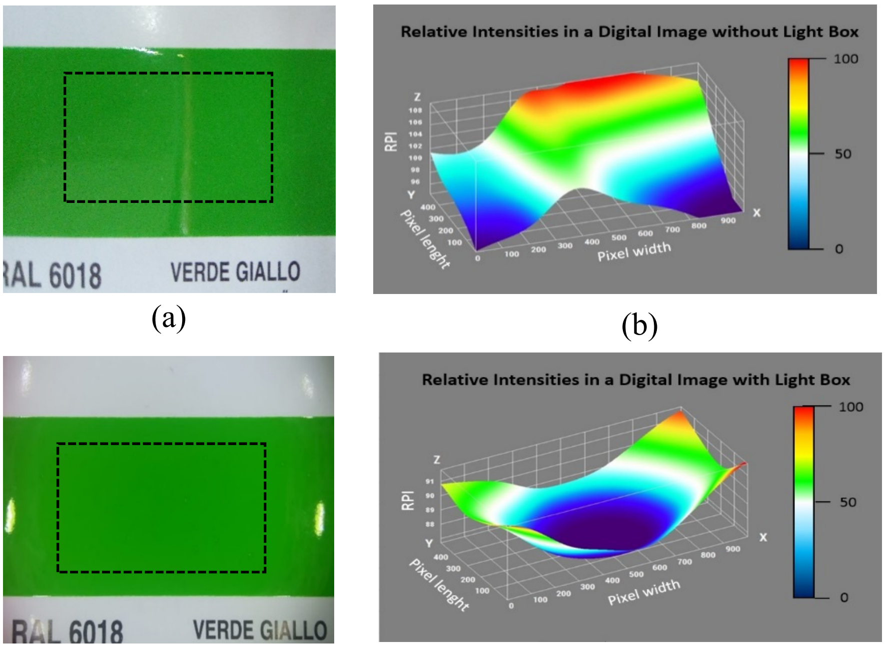


(a) (b) (c) (d)

**Figure S6.** (a) Digital 3.5 Mpixel Image in JPEG format of RAL 6018 taken in non-controlled light conditions. (b) Relative intensities in a 1000x500 pixel area of a RAL 6018 digital image in non-controlled Light conditions. (c) Digital 3.5 Mpixel Image in JPEG format of RAL 6018 taken in controlled Light conditions(Light Box). (d) Relative intensities in a 1000X500 pixel area of a RAL 6018 digital image in controlled Light conditions (Light Box).

- Finally, to evaluate the effect of external light inside the light box, an external light source was used at a distance of 20 cm from the box. The light box was completely closed except for an opening through which an optical fiber (Oceans Optics QP600-1-sR) coupled to a compact monochromator (Oceans Optics QE-65000) was inserted to measure its hermeticity (Figure S7a).The light box did not have internal lighting during the study. As shown in the Figure S7b, no signal was captured from the external light source.

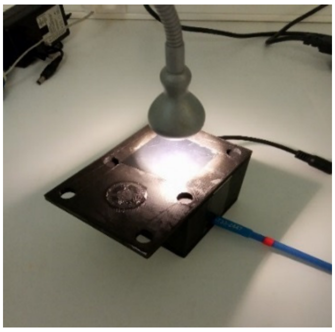
(a)

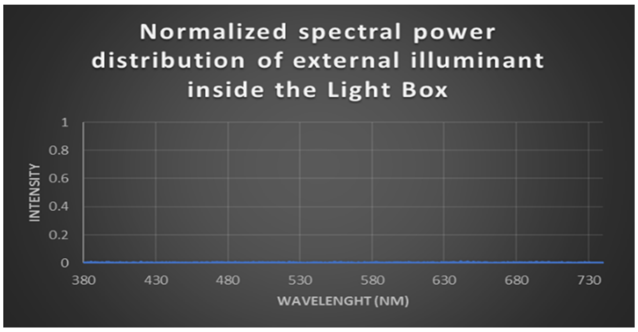

(b)

**Figure S7.** (a) Disposition of the Light source and the light box to evaluate the external light effect on color samples inside the box**.** (b) Normalized spectral power distribution plot of external illuminant inside the light box

**Supplementary Material 12:**

**CIEΔ$E_{2000}$ comparison of 55 RAL color samples between three different smartphones before/after the correction method:** A first approach, in correcting color measurements and making the comparison between devices, is to correct the initial RGB values captured by the sensor. In order to do this, each device has to be corrected with its individual and unique matrices. Once corrected, they can be compared with CIEΔ$E_{2000}$ (Table S6).

**Table S6.** Evaluation of 55 RAL color samples with the CIEΔ$E_{2000}$ (2:1:1) criterion using 3 different Smartphones with/without the correction method under controlled lighting conditions (Light Box), CI (95%)

| **Without Correction** | **ΔL** | **Δa** | **Δb** | **Δ$E_{2000}$** |
|---|---|---|---|---|
| Xiaomi Redmi 6A | 5.52 ± 0.98 | 8.01 ± 1.44 | 7.25 ± 1.38 | 5.82 ± 0.46 |
| Xiaomi Redmi 4A | 6.14 ± 0.95 | 4.55 ± 0.90 | 4.29 ± 0.95 | 4.67 ± 0.52 |
| Iphone SE | 4.05 ± 0.75 | 6.47 ± 1.38 | 5.43 ± 1.15 | 4.91 ± 0.68 |

| **With Correction** | **ΔL** | **Δa** | **Δb** | **Δ$E_{2000}$** |
|---|---|---|---|---|
| Xiaomi Redmi 6A | 1.90 ± 0.38 | 2.05 ± 0.46 | 1.93 ± 0.40 | 2.01 ± 0.22 |
| Xiaomi Redmi 4A | 2.49± 0.48 | 2.02 ± 0.43 | 2.67 ± 0.55 | 2.38 ± 0.30 |
| Iphone SE | 1.37 ± 0.29 | 2.35 ± 0.49 | 2.30 ± 0.42 | 2.02 ± 0.24 |

**Supplementary Material 13:**

**Colorimetric reaction of the Quantofix Peroxide 25 ® test strips:** These strips contain a Horseradish Peroxidase-like enzyme to catalyze the reaction and 3,3′,5,5′-Tetramethylbenzydine, that acts as the colorant of the redox reaction. The color changes produced on the strips occur between 0 to 25 mg/L of $H_2O_2$, generating a graduation of a cyan-like color. The colorimetric reaction is shown in Figure S8.

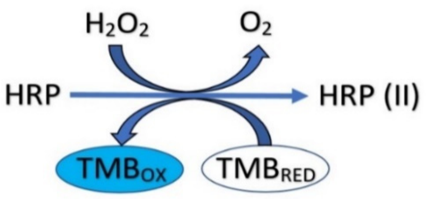


**Figure S8.** Colorimetric reaction of the Quantofix Peroxide 25 ® test strips. The oxidized form of the 3,3´,5,5´-Tetramethylbenzydine generates a cyan-like color.

**Supplementary Material 14:**

**Variability between devices in the colorimetric reaction of the Quantofix Peroxide 25 ® test strips:** Samples 1, 2, 3 and 4 were chosen for a box and whiskers plot so the variability between devices, before and after the correction method, could be noticed. The plot is shown in Figure S9.

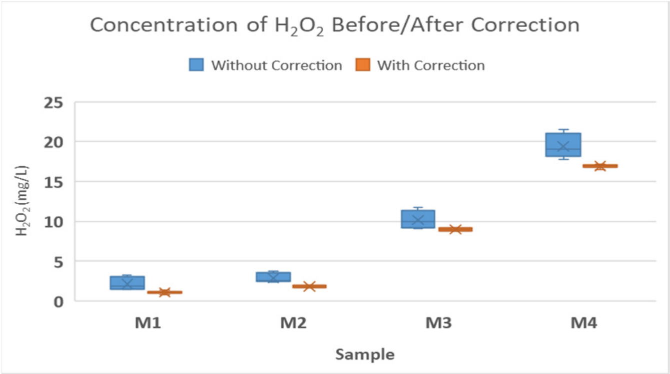


**Figure S9.** Box and Whisker Plot of four samples before and after the correction method. The plot shows that in every case the [$H_2O_2$] before the correction is higher than the after it and has more variability.

**Supplementary Material 15:**

**Dependence of the RGB measurements with the pH values:** The representation of each individual RGB value with the pH (Figure S10). It shows the color gamut changes of the PanReac AppliChem® pH strips.

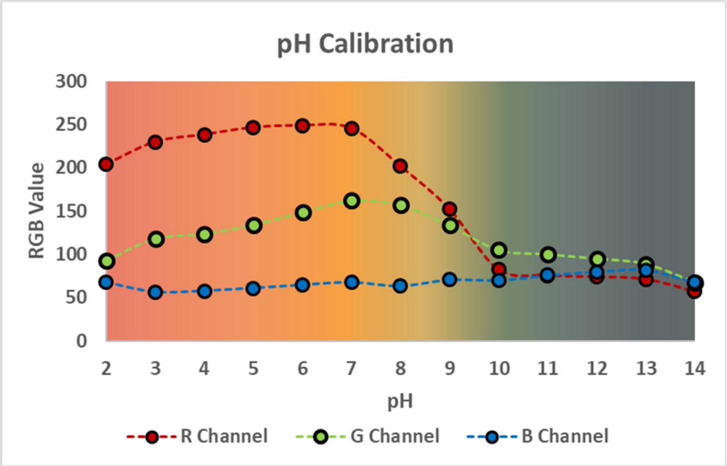


**Figure S10.** A graph of the dependence of each RGB channel with the pH. The R Channel shows better sensibility than the other channels but there are no significant differences of the R coordinate in the range 2 – 7 of pH, neither in the range 10 – 14.

**Supplementary Material 16:**

**Variability between devices in the colorimetric reaction of the PanReac AppliChem® pH strips:** Samples 1, 2 and 3 were chosen for a box and whiskers plot (Figure S11) so the variability between devices, before and after the correction method, could be noticed. Sample 4 was discarded since the initial color coordinates could not be interpolated on the calibration plot.

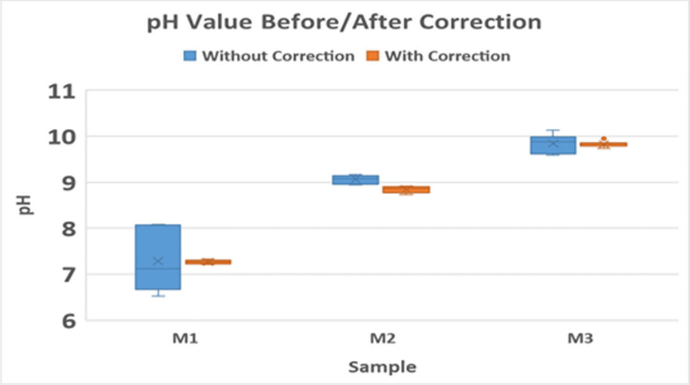


**Figure S11.** Box and Whisker Plot of three samples before and after the correction method. The plot shows that in every case the pH values before the correction has more variability.

## References


1. Furtner, U. Color processing with Bayer Mosaic sensors. 2001.
2. The Bayer Sensor Strategy. Bayer Array Image. Available online: https://www.red.com/red-101/bayer-sensor-strategy (accessed on 2 March 2022).
3. Wyszecki, G.; Stiles, W.S. *Color science: Concepts and Methods, Quantitative Data and Formulae*, 2nd Ed.; Wiley: New York, NY, USA, 1982; pp. 11–27, 51–66, 83–93, 119–154, 429–439.
4. Bruce Linbloom Website. Useful Color Equations. Available online: http://www.brucelindbloom.com/ (accessed on 2 March 2022).
5. Ibraheem, N.A.; Hasan, M.M.; Khan, R.Z.; Mishra, P.K. Understanding color models: A review. *ARPN J. Sci. Technol.* **2012**, *2*, 265–275.
6. Ford, A.; Roberts, A. *Colour Space Conversions*; Westminster University: London, UK, 1998; pp. 1–31.
7. Mokrzycki, W.S.; Tatol, M. Colour difference ∆ E-A survey. *Mach. Graph. Vis.* **2011**, *20*, 383–411.
8. Melgosa, M. CIE/ISO New Standard: CIEDE2000. In *CIEDE Workshop*; University of Leeds, Leeds, UK, 2013.
9. Holm, J.; Maier, T.; Debevec, P.; LeGendre, C.; Pines, J.; Erland, J.; Joblove, G.; Dyer, S.; Sloan, B.; di Gennaro, J.; et al. A Cinematographic Spectral Similarity Index. In Proceedings of the SMPTE 2016 Annual Technical Conference and Exhibition, Hollywood, CA, USA, 25–27 October 2016; pp. 1–36, https://doi.org/10.5594/M001680.
10. Academy Spectral Similarity Index (SSI) Calculator (BETA). Available online: http://ssi-calculator.oscars.org/ (accessed on 2 March 2022).
